# Effects of larval growth condition and water availability on desiccation resistance and its physiological basis in adult *Anopheles gambiae *sensu stricto

**DOI:** 10.1186/1475-2875-9-225

**Published:** 2010-08-07

**Authors:** Fred Aboagye-Antwi, Frédéric Tripet

**Affiliations:** 1 Centre For Applied Entomology and Parasitology, School of Life Sciences, Keele University, Keele, Staffordshire, ST5 5BG, UK

## Abstract

**Background:**

Natural populations of the malaria mosquito *Anopheles gambiae *s.s. are exposed to large seasonal and daily fluctuations in relative humidity and temperature, which makes coping with drought a crucial aspect of their ecology.

**Methods:**

To better understand natural variation in desiccation resistance in this species, the effects of variation in larval food availability and access to water as an adult on subsequent phenotypic quality and desiccation resistance of adult females of the Mopti chromosomal form were tested experimentally.

**Results:**

It was found that, under normal conditions, larval food availability and adult access to water had only small direct effects on female wet mass, dry mass, and water, glycogen and body lipid contents corrected for body size. In contrast, when females subsequently faced a strong desiccation challenge, larval food availability and adult access to water had strong carry-over effects on most measured physiological and metabolic parameters, and affected female survival. Glycogen and water content were the most used physiological reserves in relative terms, but their usage significantly depended on female phenotypic quality. Adult access to water significantly influenced the use of water and body lipid reserves, which subsequently affected desiccation resistance.

**Conclusions:**

These results demonstrate the importance of growth conditions and water availability on adult physiological status and subsequent resistance to desiccation.

## Background

The geographic range of *Anopheles gambiae *sensu stricto, the major malaria vector in sub-Saharan Africa, includes semi-arid regions characterized by strong seasonal variations in temperature, relative humidity and rainfall [1,2]. In West Africa, seasonality has been associated with changes in the relative abundance of certain chromosomal inversion arrangements thereby underlining the role of some chromosomal inversions in conferring resistance to desiccation [3]. The Mopti chromosomal form of *An. gambiae *is characterized by high frequencies of *a *and *bc *inversions which are thought to confer resistance to drought. It also predominates in drier parts of the country and increases in relative abundance during the dry season [4,5]. In contrast, the chromosomal arrangements of the Bamako and Savannah forms lack this combination of inversions. These forms are better adapted to the wetter parts of Mali and their relative abundance and geographical distribution increases in the rainy season [4,5]. Thus drought and the selection pressures associated with hydric stress (stress induced by limited water availability) are important for the evolution and maintenance of genetic polymorphism in *An. gambiae'*s populations and for determining the respective distribution and abundance of chromosomal forms [4,6].

In addition to seasonal droughts, mosquito populations in the Sahelian belt are subjected to strong daily fluctuations in temperature and relative humidity. These persistent environmental fluctuations make adaptations for coping with hydric stress and maintaining an adequate body water balance, some of the most important aspects of mosquito behaviour and physiology [7]. In contrast to an extensive body of literature focusing on those aspects in *Drosophila melanogaster *[8-16], only very few studies have experimentally studied resistance to desiccation in adult *An. gambiae *sensu lato. Using laboratory maintained colonies, Gray and Bradley [7] showed that *Anopheles arabiensis *was more desiccation resistant than its sister species *An. gambiae *s.s. In Mali, the Mopti chromosomal form is characterized by the M-type of *r*DNA and the Savanna or Bamako chromosomal forms by the S type [17]. Using offspring from wild-caught blood-fed females, Lee *et al *[6] recently demonstrated, that desiccation resistance was higher in individuals of the M molecular form than in those of the S molecular form, thereby corroborating the correlation found between desiccation resistance and the spatial and temporal distribution of chromosomal forms in that region. Finally, a recent study showed using a heterogeneous laboratory colony of the Forest chromosomal form from Cameroon which was split and selected either for the *a *inversion or the standard arrangement showed that young adults homozygote for *a *better resisted desiccation than those without the inverted arrangement [18].

In natural mosquito populations, genetic polymorphism is probably one of many factors that can possibly affect the desiccation resistance of an individual mosquito. The impact of hydric stress on an individual is also likely to depend on factors affecting its phenotypic quality - i.e. its morphological, developmental, behavioural, biochemical and physiological properties. For example, in *An. gambiae*, it has been amply demonstrated that larval growth conditions influence adult body size and condition, which in turn affects other important traits such as immune response to infection and blood meal utilization [19,20]. Similarly, larval growth conditions through their effect on teneral reserves and generally body size and condition [21,22,20], could impact the ability of adult mosquitoes to cope with desiccation [7]. Although a few studies have examined the effects of body size on fitness in mosquitoes [19-23], none of these studies sought to clarify the contribution of physiological reserves in relation to phenotypic quality and desiccation resistance in *An. gambiae *s.s. Indeed, almost all the studies investigating the mechanisms of resilience to desiccation are based on selection experiments conducted on *Drosophila *species [8,10,15,16,24].

There are three recognized physiological mechanisms by which an insect may overcome or cope with desiccation: (i) increasing water storage either in the form of bulk water (water molecules obtainable from sources other than catabolism) or metabolic water (water molecules obtainable directly from catabolism) or both, (ii) regulating water loss through respiratory and trans-cuticular transpiration, and (iii) being tolerant of water loss [16]. In Drosophilids, adaptations such as lower cuticular permeabilities and water loss rates, as well as higher dry and wet mass have all been shown to be associated with desiccation resistance [7,25-27]. Archer *et al *[16] demonstrated that increased bulk water content and lower water loss rate were the most important physiological mechanism for desiccation resistance in *Drosophila melanogaster*. Gray and Bradley [7] found that differences in bulk water content rather than water loss rates explained the higher desiccation resilience of *An. arabiensis *than its sister species *An. gambiae *s.s. Metabolic water is generated through the catabolism of glycogen and lipids. Glycogen has the ability to bind the equivalent of three to five times its mass of water and to release it during glycogenolysis [28], making it a significant source of water during desiccation stress [15,29]. Lipids can proportionally yield the highest amounts of water per unit of mass and may thus also serve as an important source of metabolic water [30]. However, lipids have so far not been associated with desiccation resistance in *Drosophila *[24,29,31].

To date, the physiological mechanisms responsible for desiccation resistance in *An. gambiae *s.s. remain to be determined. In the present study, the effect of variation in phenotypic quality and water availability on survival of females of the Mopti chromosomal form of *An. gambiae *s.s. under desiccation stress were experimentally investigated and changes in wet and dry body mass as well as water, glycogen, cuticular lipid and full-body lipid contents recorded. The results emphasize the general importance of glycogen metabolism for desiccation resistance as well as the impact of variation in phenotypic quality and water availability on resistance via their effects on body water and body lipid reserves.

## Methods

### Manipulation of food availability at the larval stage

Adult female *An. gambiae *s.s. of the Mopti strain were fed on horse blood using an artificial feeder (Hemotek membrane feeding system, Discovery workshops, UK) pre-warmed to 37°C. Eggs were laid two days post blood-feed and hatched within two days. A day later, newly emerged first instars were distributed into plastic trays (34 × 24 cm) at densities of 200 per tray in 1L of water. Eight trays were assigned to a feeding regime of 40 mg of ground fish food (Tetra werk, Mulle, Germany) twice a day (morning and evening) to yield large adults of good phenotypic quality. A second batch of eight trays received a single feed of 40 mg ground fish food each day, yielding smaller size adults of poor phenotypic quality.

### Manipulation of water availability at the adult stage

Three hundred and sixty 1-3 day old female mosquitoes from the high feeding regime treatment, referred as 'good phenotypes' and the same number of females from the low feeding regime treatment, or 'poor phenotypes' were distributed into 8 pint-size cages (45 females per cages) and assigned to two water availability treatments for 7 days. The first group was supplied with water 16 h per day (stress condition) and the second with water always (optimum condition). All mosquitoes were provided with sugar in the form of cubes *ad libitum*, kept at 65-70% relative humidity (RH) and a temperature of 26°C. The cages were organised using a 4 × 4 Latin square design and shuffled daily within replicate in order to completely eliminate confounding environmental effects in the insectary. All cages were examined daily for dead mosquitoes, which were removed and stored individually in 1.5 ml centrifuge tubes at -20°C. These procedures were followed for two successive experiments (see below). The manipulation of water availability was optimized such that it did not lead to higher mortality in stressed females during the 7 d hydric stress period of either experiment (Proportional Hazard likelihood ratios: phenotypic quality: *p *> 0.6 in both cases; hydric stress: *p *> 0.4 in both cases).

### Seven-day hydric stress experiment

In a first experiment, females from the four experimental groups (i.e. good and poor phenotypic quality and water 16 h per day (stress condition) or water always (optimum condition)) that survived till the 7th day of water availability treatment were killed by freezing and their body size (wing length from the alular notch to the distal wing margin), wet mass, dry mass and water content assessed. They were then randomly assigned to one of two groups for metabolite analyses. One group was assessed for body lipid content and the other for glycogen content (see details below).

### Hydric stress and desiccation challenge experiment

In a second experiment, which followed exactly the same methods as the first, females from the four experimental groups (i.e. good and poor phenotypic quality and water 16 h per day or water always) that survived until the 7^th ^day of water availability treatment were placed in an incubator set at 30°C and 30% relative humidity (RH) without water and sugar until death. This is referred to as 'desiccation challenge' throughout the text. The number of 'dead' mosquitoes was recorded every hour in order to establish survival curves for each experimental group. To avoid problems associated with the fast loss of water that accompanies death, mosquitoes we considered 'dead' when they were too weak to fly and could not stand on their legs. Typically, these signs indicate that death will occur within the following hour.

The same physiological and metabolite measurements were taken as in the 7 d hydric stress experiment and the same procedure was used to randomly assign females to different metabolite measurements.

### Metabolite measurements procedures

#### Wet and dry mass, water content

Dead mosquitoes were placed individually in labelled 1.5 ml microcentrifuge tubes and stored at -20°C. Each individual mosquito was weighed in a weighing boat within 24 h of being frozen, to a precision of 0.01 mg using a Sartorius^® ^electrobalance; Model 1800, Germany. Mosquitoes were then dried in an incubator at 60°C for over three hours, a duration that is adequate to ensure complete drying of samples as shown in different studies [8-10,16] and through our own preliminary tests. Dried mosquitoes were re-weighed individually and water content was calculated as the difference between the wet and dry mass [29].

#### Glycogen content

Glycogen content was measured following Van Handel's [32] method with modifications. Each sample was placed in a 1.5 ml microcentrifuge tube with 500 μl of water and ground with a pestle. The microcentrifuge tubes were boiled for 5 minutes and 100 μl from each tube was transferred to a 13×100 mm test tube. Three ml of anthrone reagent (150 mg anthrone/100 ml of 72% sulphuric acid) was then added and the tubes were incubated for 20 min in a 90°C water bath. After cooling, 200 μl of the resulting homogenate were transferred to a 96-well microplate. Caution was taken to avoid air bubbles and the outer wells of the microplate were not used to prevent external light sources from interfering with the optical density readings. Absorbance was read at 620 nm using a Labsystems Multiskan^® ^Multisoft Model 349, Finland, spectrophotometer.

For each 96-well plate, mosquito samples were assayed together with a sample of known amount of glycogen and two serial dilutions of this amount for generating duplicate standard curves. For each standard curve, 6 ml of 1 mg/ml solution of glycogen were transferred into 13×100 mm test tubes. Seven additional tubes were then created by serial dilution (50%) with distilled water. From each tube, 100 μl were transferred to 13×100 mm test tubes and 3 ml of anthrone reagent added. The resulting eight test tubes contained 32.2, 16.1, 8.0, 4.0, 2.0, 1.0, 0.5, and 0.25 μg/ml of glycogen respectively. These concentrations of glycogen were plotted against their absorbance. The standard curve that best predicted the known concentrations (best-fitted curve) was used to calculate the glycogen content of the mosquito samples. To minimize plate effects, mosquito samples from all four treatments were distributed on each 96-well plate and assayed simultaneously. Glycogen content was measured three times for each mosquito and averaged.

#### Lipid content

Following a modified version of Van Handel's method [33] for determination of total lipids in mosquitoes, female mosquitoes were dried at 60°C for at least 1hr, placed in a 1.5 ml microcentrifuge tube, and 0.5 ml of chloroform-methanol (1:1) solution was added. They were ground with a pestle and the supernatant transferred to a clean 13×100 mm test tube. The solvent was evaporated by placing the tubes in a heating block at 90°C for 20 min, after which 2 ml of sulfuric acid was added and the tube re-heated for 10 min. On cooling, 5 ml of vanillin reagent (600 mg of vanillin/100 ml distilled water and 400 ml of 85% phosphoric acid) was added to each tube and the reddish coloration allowed to develop for 5 min. At that stage, 200 μl of the resultant solution of each sample were transferred into a well of a 96-well microplate for absorbance reading. The plates were read at 492 nm in a Multiskan^® ^Multisoft Model 349 spectrophotometer (Labsystems, Finland). For each plate, duplicated standard curves were generated by preparing two sets of 25, 50, 100, 150, 200, 250, 300, 350 and 400 μl, of the standard solution (1 mg vegetable oil in 1 ml of chloroform), evaporating the solvent and measuring their absorbance as described above. The curve with the best fit was used to infer lipid contents.

### Statistical analyses

Because of the known allometric relationship between mosquito body (or metabolite) mass and body size, and because of the expected effect of larval food availability on adult body size, all physiological and metabolic parameters were corrected for body size (here measured as wing length). Body (or metabolite) mass typically linearly increases with the third power of wing length because it correlates strongly with insect volume (discussed in [34]). Thus, regressing log(body mass) over log(wing length) gives a slope, or allometric coefficient, equal or close to 3 (hence the 3^rd ^power of wing length) [35]. Indeed as observed in some other studies [21,34], most of the physiological and metabolite measurements in the control group of this study very closely followed this allometry (e.g. wet and dry mass: slopes = 2.98 and 3.01 respectively, water content and glycogen content: 3.00 and 3.6). Consequently, we corrected for body size by dividing their value by the third power of wing length (mm^3^). It is important to note that other body size corrections sometimes used in ecological studies such as: (1) using wing length as a covariate in multivariate analyses, or (2) conducting all analyses on the residuals of a regression of metabolite parameters on wing length, could not be used in this study as forcing a single regression line through the pooled data of the four experimental groups assumes that all groups have equal constant and slope and this would effectively prevent us from testing true experimental treatment effects. It should also be noted that using dry mass to correct metabolite measurements as is often done in physiological studies was again unsatisfactory, as changes in metabolite and dry mass often co-varied in relation to experimental treatments, hence dividing one by the other simply obliterated treatment effects. All results and discussion focus on wet and dry mass, and water, glycogen and lipid content corrected for body size unless stated otherwise.

All statistical analyses were performed using the software JMP7.02 (SAS Institute, Inc). All data was checked for normality and heteroscedasticity and analyzed accordingly using parametric or non-parametric procedures. The effects of phenotypic quality and hydric stress on the differences in physiological and metabolic parameters between the two experiments were examined through General Linear Models which gave results consistent with the analyses of physiological and metabolic parameters at the end of the desiccation challenge. Because the two experiments represent independent datasets, those confirmatory results were taken with caution and the analyses are not presented here.

## Results

### Larval nutrition and adult body size

For both experiments limiting resources at the larval stage significantly affected adult female body size (wing length) resulting in distinct categories of phenotypic quality. Poor phenotypes measured an average 2.94 mm (2.92-2.95CI) and good phenotypes an average 3.25 mm (3.23-3.27CI) in the seven-day hydric stress experiment (*t*-Test: n = 410, *t *= 21.8, *p *< 0.001). In the 7 d hydric stress followed by desiccation challenge experiment poor phenotypes measured on average 2.86 mm (2.83-2.89CI) and good ones 3.20 mm (3.17-3.24CI) (*t*-Test: n = 223, *t *= 15.7, *p *< 0.001).

### Effects of phenotypic quality and hydric stress on survival

**Figure 1:**
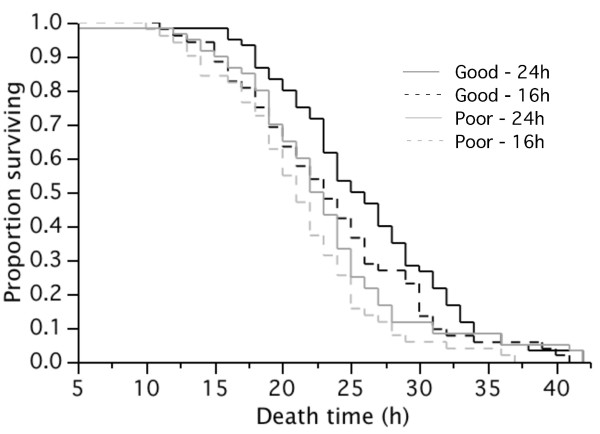
**Effect of larval nutritional stress and water availability on survival**. Effects of larval food availability at the larval stage and hydric stress at the adult stage on the survival (h) of female mosquitoes subsequently challenged with constant desiccation. Manipulation of larval food availability resulted in female of 'Good' and 'Poor' phenotypic quality, and females were provided with constant access to water (24 h) or for 16 h per day only (16 h).

The survival of adult females under constant desiccation (i.e. 7 d hydric stress and desiccation challenge exp.) was significantly affected by their phenotypic quality, and water availability during the 7 d preceding the challenge but there was no interaction between the two factors (Proportional Hazard log-likelihood ratios: n = 223, phenotypic quality, *χ*^2 ^= 7.2, *p *= 0.007; hydric stress: *χ*^2 ^= 5.3, *p *= 0.022; interaction N.S.). Females from the high phenotypic quality group with constant access to water survived on average for 26.0 h (24.2-27.7CI) and those with limited access for 23.6 h (21.7-25.5CI). Females from the low phenotypic quality group survived for an average of 23.0 h (± 6.9SD) with constant access to water and 21.2 h (19.6-22.8CI) under hydric stress (Figure 1).

### Seven-day hydric stress experiment

#### Effects of phenotypic quality and hydric stress on physiological status

**Figure 2:**
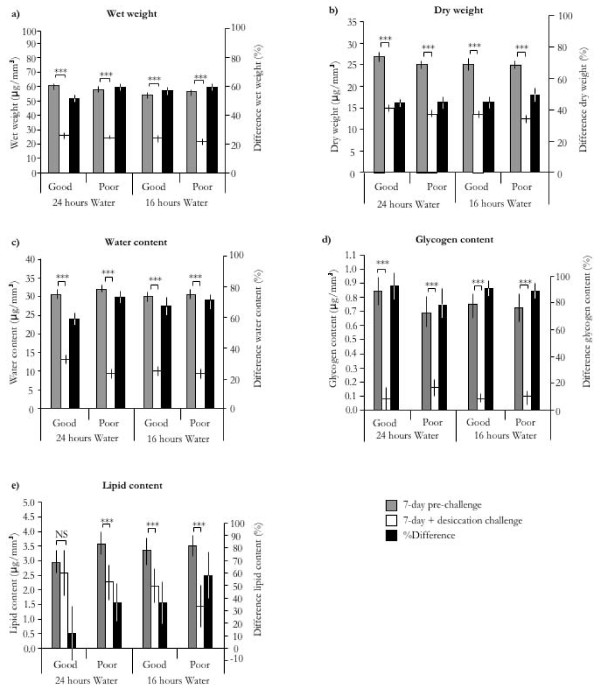
**a-e - Direct and carry-over effects of phenotypic quality and water availability on physiological and metabolic parameters**. Fig. 2a-e. Effects of mosquito phenotypic quality induced by larval nutritional stress and water availability on 5 physiological and metabolic parameters of females at the end of the 7-day hydric stress experiment (grey bars), the end of 7 d + desiccation challenge experiments (white bars), and the percentage difference between the two experiments (black bars - right axis). All parameters were corrected for body size. Confidence intervals are indicated and *P*-values for Mann-Whitney tests comparing parameters between the two experiments in each experimental groups are shown as * P < 0.05, ** P < 0.01, *** *P *< 0.001, NS not significant.

The amount of food available at the larval stage significantly affected all physiological and metabolite parameters through its effect on body size (*t*-Test or Mann-Whitney: n = 128-410, *p *< 0.001 in all cases)(for raw data see Additional Table 1). Therefore, all subsequent analyses were conducted on the data corrected for body size (see methods)(Table 1). Overall, the experimental manipulation of the amount of larval feeding and adult access to water had small and non-significant effects on the adult female stage and explained minor fractions of the parameters' variance (Table 2, Figure 2a-e). Water availability affected the wet mass of adult females resulting in significantly lighter stressed females. There were no significant interactions between the level of nutritional stress at the larval stage and hydric stress as an adult on any of the parameters examined (Table 2).

**Table 1:** Direct and carry-over effects of phenotypic quality and water availability

Experiment	Phenotype	Water availability	Wet mass	Dry mass	Water content	Glycogen content	Lipid content
**7 d hydric stress**	Good	24 h	57.4 (56.02-58.8)	26.8 (25.9-27.7	30.6 (29.6-31.6)	0.86 (0.74-0.98)	2.95 (2.62-3.30)
		16 h	54.6 (53.0-56.2)	25.1 (24.2-26.0)	29.5 (28.3-30.7)	0.75 (0.62-0.88)	3.34 (3.06-3.62)
	Poor	24 h	56.6 (54.9-58.3)	25.1 (14.1-26.0)	31.5 (30.4-32.6)	0.70 (0.62-0.78)	3.36 (3.26-3.89)
		16 h	55.5 (53.4-57.5)	25.2 (24.2-26.1)	30.3 (28.8-31.8)	0.74 (0.68-0.80)	3.52 (3.08-3.40)
**7 d hydric stress + Desiccation challenge**	Good	24 h	27.8 (26.4-29.2)	15.1 (14-4-15.8)	12.6 (11.3-14.0)	0.08 (0.04-0.12)	2.61 (1.89-3.33)
		16 h	23.4 (22.0-24.8)	13.8 (13.0-14.6)	9.6 (8.6-10.7)	0.07 (0.03-0.11)	2.14 (1.16-2.69)
	Poor	24 h	23.0 (21.8-24.3)	14.1 (13.4-14.8)	9.0 (7.8-10.0)	0.15 (0.06-0.24)	2.28 (1.65-2.90)
		16 h	22.0 (20.4-23.4)	12.9 (12.1-13.7)	9.1 (7.7-10.4)	0.09 (0.05-0.13)	1.48 (0.78-2.19)

Effects of phenotypic quality (good and poor) and water availability (24 h access and access limited to 16 h) on physiological and metabolite parameters corrected for body size (μg/mm^3 ^(CI)) in adult *An. gambiae *females at the end of the 7 d hydric stress experiment, and of the 7 d hydric stress followed by desiccation challenge experiment.

**Table 2:** Statistical analysis of direct effects of phenotypic quality and water availability

Parameter	Sample size (n)	%Variance explained (*r*^2^)	Phenotypic quality ***T*-ratio or *Z***^**†**^	Hydric stress ***T*-ratio or *Z***^**†**^	Interaction *T*-ratio
Wet mass	410	0.013	0.00	-2.33 *	NS
Dry mass	410	0.015	-1.87	-1.76	NS
Water content	410	0.014	1.44	-1.93	NS
Glycogen content^†^	130	-	1.10	1.22	-
Lipid content	128	0.048	2.32*	0.97	NS

† General linear models were used to analyze parametric data and 2 Mann-Whitney's tests were used to analyse Glycogen's non-normally distributed data (interaction not tested).

Statistical analyses of the effects of female phenotypic quality and water availability on physiological and metabolite parameters at the end of the 7 d hydric stress manipulation period. All parameters were corrected for body size (see methods for details). *P*-values are * P < 0.05, ** P < 0.01, *** *P *< 0.001, NS not significant.

### Hydric stress and desiccation challenge experiment

#### Physiological and metabolic reserve usage

Females that were subjected to the desiccation challenges lost considerable amounts of body mass. Wet mass decreased by 31.9 μg/mm^3 ^(31.2-32.6CI) equivalent to a 57.0% (55.7-58.2CI) decrease and dry mass by 11.6 μg/mm^3 ^(11.2-11.9CI) or 45.2% (43.8-46.7CI) (paired t-Test: *p *< 0.05 in both cases) (Figure 2a-b).

Comparing metabolic parameters between adult females at the end of the 7 d hydric stress experiment and females at the end of the desiccation challenge experiment revealed a significant depletion of all metabolites (paired t-Test or Wilcoxon signed-rank test: *p *< 0.05 in all cases). The largest absolute difference was predictably found in water content with a 20.4 μg/mm^3 ^(19.7-21.0CI) decrease, followed by lipids 1.28 μg/mm^3 ^(0.96-1.60CI), and glycogen 0.67 μg/mm^3 ^(0.63-0.70CI) (Figure 2c-e).

In relative terms, the largest difference however was that observed in glycogen reserves, which diminished by 86.5% (82.4-90.7CI) across all experimental groups following the desiccation challenge (Figure 2d). Water content was proportionally the next most depleted metabolite at 66.7% (64.7-68.8CI) (Figure 2c), followed by lipids, 37.1% (27.7-46.5CI)(Figure 2e).

#### Effects of phenotypic quality and hydric stress on physiological status

Constant desiccation until death resulted in stark differences in wet and dry mass and the amount of remaining metabolite reserves between the four experimental groups. Nutritional stress at the larval stage had strong carry-over effects on the proportion of physiological and metabolite reserves at death following the desiccation challenge (Table 3, Figure 2a-e). Poorer phenotypes were significantly lighter (wet and dry mass Figure 2a,b) and died with proportionally much lower water content (Figure 2c). Interestingly poor phenotypes ended up with significantly larger glycogen reserves (Figure 2d). In contrast, phenotypic quality had no significant effect on body lipid contents used (Figure 2e).

**Table 3:** Statistical analysis of carry-over effects of phenotypic quality and water availability

Parameter	Sample size (n)	%Variance explained (*r*^2^)	Phenotypic quality ***T*-ratio or *Z***^**†**^	Hydric stress ***T*-ratio or *Z***^**†**^	Interaction *T*-ratio
Wet mass	223	0.168	-4.55 ***	-3.98 ***	2.43 *
Dry mass	223	0.079	-2.70 **	-3.44 ***	NS
Water content	223	0.103	-3.45 ***	-2.36 *	2.54 *
Glycogen content^†^	75	-	3.63 ***	-1.07	-
Lipid content	74	0.074	-1.64	-2.06 *	NS

† General linear models were used to analyze parametric data and 2 Mann-Whitney's tests were used to analyse glycogen due to non-normally distributed data (interaction not tested).

Statistical analyses of the effects of female phenotypic quality and water availability on physiological and metabolite parameters at the end of the desiccation challenge. All parameters were corrected for body size (see methods for details). *P*-values are * P < 0.05, ** P < 0.01, *** *P *< 0.001, NS not significant.

Water availability significantly affected all parameters except for glycogen levels (Figure 2a-e). Adult mosquitoes with 16 h access to water ended up with significantly smaller water and lipid reserves and died having particularly low wet and dry mass compared to those supplied with constant access to water (Figure 2a,b). Phenotypic quality and hydric stress had significant interactive effects on wet mass and water content (Table 3).

## Discussion

### Effects of phenotypic quality and hydric stress on survival

This study demonstrates the importance of food availability at the larval stage and water availability at the adult stage on the ability of adult female mosquitoes to cope with desiccation stress. Mosquitoes larvae reared with food *ad libitum *gave rise to larger, but more importantly, better phenotypes that proportionally coped much better with desiccation stress as female adults. Similarly, adult females provided with constant access to water showed much greater resistance to desiccation than those raised with limited water availability.

This is the first study to experimentally test the effects of phenotypic quality and water availability on subsequent resistance to desiccation and associated physiological and metabolic changes. In the field, mosquitoes rarely have constant access to water and water availability changes seasonally in many habitats, thus they must adjust their behaviour and physiology to such changing conditions. The desiccation challenge in the second experiment was used as a common physiological assay to compare the extent of metabolic reserves amongst individuals but it was also used for simulating what would be a very strong drought-induced selection pressure. In retrospect, the desiccation challenge proved critical for understanding the strong carry-over effects of larval food deprivation and hydric stress at the imaginal stage on survival and metabolite usage of adult mosquitoes.

The data on adult desiccation resistance presented here is in agreement with previous studies in Dipterans. In *Aedes aegypti*, Mogi *et al *[36] showed that the wing length of females positively correlated with their resistance to a desiccation challenge, and a similar relationship was shown in *Drosophila *[37]. Mogi *et al *[36] also demonstrated that the survival of *Ae. aegypti *and *Aedes albopictus *adults experimentally challenged with constant desiccation increased when they had access to water; however carry-over effects were not tested in that study.

### Physiological response to hydric stress and variation in phenotypic quality

The importance of physiological status and metabolic reserves for desiccation resistance in drosophilids has been amply reported [10,16,37,38]. Surprisingly small effects of larval food deprivation and access to water on the physiological (wet and dry mass) and metabolic parameters (water, glycogen, and body lipid contents) measured at the end of the 7-day hydric stress period (all corrected for body size) were found. Although most of these parameters were predictably higher in good phenotypes and in females with constant access to water, most of these effects were non-significant despite large sample sizes. Wet mass was the only parameter significantly higher in non-stressed females, a result that could be explained by all of the above-mentioned subtle effects adding-up to significantly affect wet mass. There was no significant difference in water content, suggesting that mosquitoes reared under limited water condition can compensate for water loss during the drought periods by drinking more when water becomes available [28].

It is known that mosquitoes starved at the larval stage emerge with proportionally lower body condition as evidenced by low teneral reserves [20-22,39]. Here, small and large females did not significantly differ in dry mass and glycogen content, suggesting that small females almost completely made up for their expected initial difference in energy reserves through increased sugar feeding and water consumption. We also found that females of poor phenotypic quality accumulated significantly more total body lipid reserves than females of high phenotypic quality. In *An. gambiae *smaller females have been shown to be more likely to acquire an extra blood meal before initiating their gonotrophic cycle in order to improve their body condition [20]. Similarly, small *An. albimanus *females use the first blood meal for the synthesis of maternal lipid and protein reserves instead of oogenenesis, thereby compensating for their low metabolite reserves [21]. These results confirm earlier findings that sugar-feeding may play an important role in building-up metabolic reserves, including lipids in Anophelines before or between bloodmeals [22]. This has also been shown in Aedines, particularly in females with lesser teneral reserves [40,41].

### Physiological and metabolic changes under prolonged desiccation

The pattern of differences in physiological and metabolic parameters between the experiment with and without desiccation challenge offer interesting insights on the relative importance of desiccation resistance mechanisms in the Mopti form of *An. gambiae*. The estimates of glycogen and lipid contents before and after the desiccation challenge were in the same order of magnitude as those measured in *Ae. aegypti *and *Aedes vexans *before and after starvation [32,33,42]. Lipid estimates measured before desiccation were also comparable to other such estimates measured in *An. gambiae *[7]. In contrast, estimates of water content were somewhat lower than those reported for *An. gambiae *(55% versus 65% of total body mass) suggesting that the lower ambient humidity used to maintain females during the 7 d hydric stress period (65-70% versus 80%) may have directly affected their water content.

The most drastic relative change in metabolite observed in females subjected to desiccation until death was the strong decrease observed in glycogen level, which was nearly completely used up. In *Drosophila*, glycogen seems to be the major metabolite utilized in desiccation resistance and mass loss has been attributed to its catabolism [15,43,44]. Water bound to glycogen is released during glycogenolysis and it is this ability to bind up water that makes glycogen relevant for desiccation resistance [29]. The second largest relative difference and largest absolute decrease observed in desiccation-challenged females was in water content, a difference that can be explained by respiration and cuticular transpiration. These processes may also be responsible for the loss of water that would be generated via glycogen catabolism. Body lipids were also significantly used and although that difference was less dramatic in relative terms than those observed in glycogen and water contents, the absolute difference observed in lipids was larger than that in glycogen because lipids make for a larger proportion of the total body mass. Metabolisation of body lipids is known as a means of replenishing diminishing water reserves when bulk water and glycogenolysis-derived water sources have been exhausted at least in some insects [15,30,45,46] although its direct role in relation to desiccation in Drosophila is not clear cut (reviewed in [44,47]). Interestingly, Gray *et al *[18] recently showed that aside from differences in body water content at emergence and in young adults, individuals from 2 homokaryotypic populations selected either for the *a *inversion or the standard inversion also differed in their relative investment in glycogen and lipid reserves when acclimated to drier conditions.

It is important to note that since glycogen and lipids represent ~15% of total dry mass their catabolism alone cannot explain the observed 45% decrease in dry mass observed in females challenged by desiccation. This suggests that a number of other metabolites, such as sugars (other than glycogen), as well as salts, amino acids, peptides, and larger proteins may either be lost or metabolized during the desiccation challenge and this would contribute to mass loss. In addition, the catabolism of some of those compounds, such as proteins, could potentially contribute to enhanced desiccation resistance and this remains to be explored experimentally. It is noteworthy that there is a fine line between what constitutes a starvation and a desiccation challenge, since the later typically involves the removal of both food and water. This may blur interpretations, particularly in experiments with Anophelines such as this one, as there is evidence that they can mobilize not only substantial portions of their carbohydrates and lipid reserves but also up to 53% of their proteins within 1-2 days of starvation [22].

### Carry-over effects of hydric stress and phenotypic quality on desiccation resistance

Contrary to what was found after the 7-day hydric stress experiment, which revealed only subtle differences in physiological and metabolic parameters of adult females, the second experiment which included a challenge with constant desiccation until death revealed strong carry-over effects of larval food deprivation and experimental manipulation of water availability. Food availability at the larval stage significantly determined wet and dry mass, and water content, which were all proportionally significantly higher in good phenotypic quality female. The most likely mechanism explaining the large differences found in wet mass and water content between good and poor phenotypes faced with desiccation is the allometric relationship between body mass (or volume) and transpiration. As the surface to volume ratio of females decreases with increasing body size, good phenotype females will proportionally lose less water through respiration and cuticular transpiration than smaller individuals and consequently will use up less of their water reserves [15,28,37]. The significant interaction found between the effects of larval nutritional stress and adult access to water lends further support to this hypothesis as one would expect hydric stress to have a stronger carry-over effects on females of poor phenotypic quality than on good ones in case of allometric transpirational water loss.

Surprisingly, glycogen levels at the time of death were higher in poor phenotypic quality females than in good ones and did not contribute to their overall lower wet and dry mass. This experimental group had the lowest water content (40.2% of wet mass) and might have past a critical threshold of bulk water after which metabolic water sources such as glycogenolysis could not insure viability. Although lipids tended to be lower in smaller females this effect was not significant. Since it was shown in the first experiment that poor phenotypes accumulated significantly more lipids than good ones, this suggests that they used those extra reserves to survive the desiccation challenge. This pattern of heightened lipid storage and use in individuals of lower phenotypic quality in response to desiccation is thus consistent with what we know of compensatory lipid build-up in mosquitoes [21,22,34] but contrasts with what has generally been described in *Drosophila *[43,44]. The overall higher dry mass in females of good phenotypic quality can thus be partially explained by the fact that lipids, which made for a much larger fraction of body mass than glycogen, tended to remain in higher amounts in females of good phenotypic quality (see Figure 2).

Hydric stress prior to the desiccation challenge had strong carry-over effects on all physiological and metabolic parameters of adult females except for glycogen content. The lack of difference in glycogen between females that had had limited and constant access to water may be due to the fact that females used up those reserves almost completely and would thus confirm glycogenolysis as a limited but important source of water. The significant difference observed in wet mass between females with partial and full access to water prior to the challenge can be attributed to the combined differences in water and lipid contents whilst the difference in dry mass may be linked to differences in lipid content alone. Indeed, the magnitudes of those differences add-up very well (see Table 1). However, and as discussed above, water, glycogen and lipid content alone only partly explain the drastic overall loss in body mass experienced by females that went through the desiccation challenge and proteolysis would be the simplest explanation to explain such discrepancy.

Insects adapted to arid environments also commonly exhibit reduced cuticular water loss due to a higher content of cuticular lipids [12,13,48] and possibly through changes in cuticular lipid composition [12,13]. Thus the possibility that phenotypic quality or hydric stress prior to the desiccation challenge affected patterns of water loss in our experiment cannot be dismissed and would warrant further studies.

## Conclusions

This study demonstrate the importance of larval growth conditions and access to water on the physiological state of adult females of the Mopti chromosomal form and how this in turn affects their ability to cope with desiccation. The results provide evidence that bulk water and the catabolism of glycogen and lipids are critical for desiccation resistance in *An. gambiae*, but that their respective importance and usage is dependent on phenotypic quality. Similarly the use of bulk water and lipids differed according to female access to water prior to the desiccation stimulus. These results thus emphasize the variety and complexity of the adaptations to desiccation characterizing *An. gambiae *populations in much of their sub-Saharan distribution and which contribute to making this species one of the most important for malaria transmission [1].

## Competing interests

The authors declare that they have no competing interests.

## Authors' contributions

FT and FAA planned and designed the experiment, FAA conducted the experiments, FT and FAA conducted the data analyses. FAA and FT wrote the manuscript.

All authors read and approved the final manuscript.

## Supplementary Material

Additional file 1 **Direct effects of phenotypic quality and water availability (data not corrected for body size)**. Effect of phenotypic quality (good and poor) and water availability (24 h access and access limited to 16 h) on the raw values of physiological and metabolic parameters (mg/fly (CI) - no size correction) on adult *An. gambiae *females at the end of the 7 d hydric stress experiment, and of the 7 d hydric stress followed by desiccation challenge experiment. Click here for file
