# Supplementary material for: Effects of larval growth condition and water availability on desiccation resistance and its physiological basis in adult Anopheles gambiae sensu stricto
Source: Malar J. 2010 Aug 7;9:225. doi: 10.1186/1475-2875-9-225 (PMC2922302; doi:10.1186/1475-2875-9-225)
Supplement: Additional file 1 — Direct effects of phenotypic quality and water availability (data not corrected for body size). Effect of phenotypic quality (good and poor) and water availability (24 h access and access limited to 16 h) on the raw values of physiological and metabolic parameters (mg/fly (CI) - no size correction) on adult An. gambiae females at the end of the 7 d hydric stress experiment, and of the 7 d hydric stress followed by desiccation challenge experiment. [file 1475-2875-9-225-S1.DOC]

**Additional file 1**.

| **Experiment** | **Phenotype** | **Water availability** | **Wet mass** | **Dry mass** | **Water**  **content** | **Glycogen**  **content** | **Lipid content** |
| --- | --- | --- | --- | --- | --- | --- | --- |
| **7d hydric stress** | Good | 24h | 1.97  (1.90-2.04) | 0.92  (0.88-0.96) | 1.05  (1.01-1.10) | 0.0295  (0.0247-0.0344) | 0.097  (0.085-0.109) |
|  |  | 16h | 1.91  (1.82-1.99) | 0.88  (0.83-0.92) | 1.03  (0.98-1.08) | 0.0277  (0.0221-0.0333) | 0.109  (0.099-0.118) |
|  | Poor | 24h | 1.47  (1.41-1.47) | 0.65  (0.62-0.67) | 0.82  (0.78-0.86) | 0.0192  (0.0170-0.0214) | 0.086  (0.078-0.095) |
|  |  | 16h | 1.39  (1.33-1.45 | 0.63  (0.60-0.66) | 0.76  (0.72-0.80) | 0.0197  (0.0180-0.0225) | 0.081  (0.071-0.091) |
| **7d hydric stress + Desiccation challenge** | Good | 24h | 0.94  (0.88-0.94) | 0.51  (0.48-0.54) | 0.43  (0.378-0.48) | 0.0027  (0.0013-0.0041) | 0.075  (0.054-0.096) |
|  |  | 16h | 0.76  (0.70-0.82) | 0.45  (0.41-0.48) | 0.32  (0.28-0.25) | 0.0026  (0.0012-0.0040) | 0.066  (0.048-0.084) |
|  | Poor | 24h | 0.54  (0.50-0.57) | 0.33  (0.31-0.35) | 0.21  (0.18-0.24) | 0.0033  (0.0014-0.0052) | 0.055  (0.040-0.069) |
|  |  | 16h | 0.51  (0.48-0.55) | 0.30  (0.28-0.320 | 0.21  (0.18-0.25) | 0.0022  (0.0013-0.0029) | 0.036  (0.020-0.051) |
